# Modifiable Lifestyle Factors Associated with Cognitive Recovery from MCI: A 12-Year Longitudinal Study

**DOI:** 10.1093/geroni/igab046.2652

**Published:** 2021-12-17

**Authors:** Bingyu Li

**Affiliations:** Shenzhen University, Shenzhen, Guangdong, China (People's Republic)

## Abstract

Introduction: Many studies have investigated the risk factors associated with progression from mild cognitive impairment (MCI) to cognitive impairment, while it is unclear which lifestyle factors are associated with cognitive recovery among those who have mild cognitive impairment. Methods: The study includes 7,422 participants above 65 years old with MCI from The Chinese Longitudinal Healthy Longevity Survey (CLHLS). Cox regression analysis was adopted to investigate the association between cognitive recovery and selected lifestyle factors. LASSO was applied to select the variables. Results: Daily consumption of fresh fruits is associated with higher possibility of cognitive recovery (HR: 1.28, 95% CI: 1.15-1.42) while daily consumption of meat show opposite influence (HR: 0.90, 95% CI: 0.80-0.99). Smoking (HR: 0.99, 95% CI: 0.98-1.00) and alcohol consumption (HR: 1.00, 95% CI: 0.99-1.00) are both negatively associated with cognitive recovery. Daily engagement in reading (HR: 1.24, 95% CI: 1.00-1.54), housework (HR: 1.21, 95% CI: 1.08-1.35) as well as mahjong and other card games (HR: 1.23, 95% CI: 1.08-1.39) are associated with higher possibility of cognitive recovery. Conclusion: This study has identified important modifiable lifestyle factors associated with natural cognitive recovery from MCI. The findings have considerable implications for dementia prevention.

